# Ultradian and Infradian Rhythms in the Dynamic of Testosterone Concentration in the Serum of the White-Breasted Hedgehog *Erinaceus roumanicus*

**DOI:** 10.1038/s41598-020-63399-5

**Published:** 2020-04-14

**Authors:** Marina ;V. Rutovskaya, Anna M. Kosyreva, Mikhail E. Diatroptov

**Affiliations:** 1 A. N. Severtsov Institute of Ecology and Evolution Russian academy of Science, Moscow, Russia; 2 Department of Immunomorphology of Inflammation, Research Institute of Human Morphology, Moscow, Russia

**Keywords:** Steroid hormones, Oscillators

## Abstract

The aim of the study was to identify ultradian (intraday) and infradian (multi-day) rhythms in the dynamics of testosterone concentration in the blood serum of white-breasted hedgehogs. Blood sampling was performed from the femoral veins of 12 male hedgehogs. We found ultradian rhythms of testosterone on both sampling dates—March 7–8 (a day length of 11 hours and 15 minutes) and May 10–11 (a day length of 16 hours). An 8-hour rhythm of testosterone concentration has been established. The acrophases were at the same times in both photoperiods and thus independent of sunset times. The study of the infradian rhythms of testosterone was daily carried out on May 22—June 3, at 07:40 to 08:50 and from June 27 to July 7, at 16:15–16:50. It revealed an infradian rhythm of the testosterone concentration with a period of 4-days in both the morning and the evening sampling. According to our previous investigation, the infradian rhythms of testosterone among individual hedgehogs, rodents and primates have the same period. That indicates the common mechanisms for their formation. In case of experimental studies, the phase of ultradian and infradian biorhythms will need to be taken into account because the testosterone concentration in acrophase is 2–4 times higher than in bathyphase.

## Introduction

The dynamics of physiological indicators of the organism have not only circadian but also ultradian and infradian rhythms^1–4^. Unlike circadian biorhythms, the mechanisms of ultradian and infradian biorhythms formation is not yet understood. However, there are facts pointing to the relationship of these biorhythms with quasi-rhythmic fluctuations of environmental factors, which differ from light/dark mode^5,6^.

Recently 2–6 hourly fluctuations of various physiological indicators, in particular, motor activity, sleep, nutrition, body temperature and serum hormone levels have been studied^7–9^. It was shown that the activity of the hypothalamic-pituitary-adrenal axis has both circadian and ultradian rhythms^9^. Ultradian glucocorticoid hormone secretion rhythms are important for normal gene transcription, regulation of metabolism, inflammation, (cognition, memory) and reactions to stress. However, the nervous and endocrine processes that determine these rhythms are not fully understood^9^.

It was shown that the identified dopaminergic oscillator has a period of about 4 hours and, along with the circadian oscillator, controls the rest/activity cycle. Furthermore, the disruption of the dopamine transporter gene leads to an increase in the period of locomotor ultradian rhythms in mice, and the striatal dopamine levels fluctuate in synchrony with the ultradian activity cycles^10^.

It is well-known that there are circaseptan (about-weekly) and circasemiseptan (about-half- weekly) infradian biorhythms of the glucocorticoid hormones concentration, melatonin level, mitotic activity, sodium excretion, heart rate variability, etc.^11–15^. It is assumed that these rhythms, as well as circadian, are self-sustaining and have external synchronisers that have not yet been established^1,16^. The concentration of testosterone in the blood serum has very pronounced (high-amplitude) fluctuations as in the ultradian and infradian ranges^17,18^. This suggests, that the fluctuations of this hormone are necessary for the normal functioning of the testes^3^. Moreover, the infradian rhythm of testosterone might provide synchronisation of reproductive behaviour in male and female individuals.

The important question is whether the peaks in testosterone concentration relate to the time of sunrise/sunset. Given the fact that hedgehogs begin their activity with the onset of twilight, it is logical to assume that the acrophase of testosterone concentration depends on the time of twilight starting. However, the results of experiments performed on laboratory animals (rabbits and rats) indicate the absence of a connection between the phase of the ultradian rhythms of testosterone and the light/dark mode^19,20^. Which pattern is shown by wild hedgehogs is unknown and is investigated in this study.

One of the representatives of wild hedgehogs in Central Russia is the white-breasted hedgehog, *Erinaceus roumanicus*. The males have relatively large sizes: body length is 253–285 mm, tail is 13–33 mm^21^, and the weight of the animals varies greatly according to the seasons of the year and range from 0.8 to 1.8 kg. The maximum weight occurs typically before hibernation^22^. Hedgehogs (genus *Erinaceus*) are classic hibernators. Hibernation is characterised by a decrease in body temperature, a slowing of the heartbeat (from 128–210 to 2–12 beats per min), respiratory depression (from 50 to 4–5 respiratory acts per minute), decrease in the intensity of all biochemical processes and a stop of spermatogenesis^23^. In mid-April and May female hedgehogs (*Erinaceus europaeus*) have clearly spontaneous ovulation^24^. There may be several cycles of ovulation, and as a result of the first mating, a pseudo-pregnancy often forms; after, a new ovulation occurs on the 7th–10th day. The exact period of the oestrus cycle of *Erinaceus europaeus* is unknown. Subsequent mating leads to pregnancy, of which the duration is about 1 month^24^. There are 2–10 pups in the litter and most often 5–6. Females in the north have usually 1 litter per year^21^.

The concentration of testosterone in white-breasted hedgehogs has a pronounced seasonal dynamic. Maximum values are observed immediately after the awakening of animals in March–April. Then the concentration of this hormone gradually decreases, and from August, it drops sharply to trace concentrations^25^.

Study of infradian and ultradian rhythms in hormone concentrations were mostly carried out on humans and laboratory animals, mainly rodents and lagomorphs. The study of rhythm in the dynamics of testosterone levels in hedgehogs is important from both theoretical and practical points of view. The order *Insectivora* (*Eulipotyphla*), to which the hedgehog family (*Erinaceidae*) belongs, is included in the *Laurasiatheria* superorder, and it is basal for the predatory, ungulate, cetacean and bat orders of the same superorder. On the other hand, the superorder *Laurasiatheria* is the sister for superorder *Euarchontoglires*, which, in particular, includes rodents and primates^26^. The separation of these superorders occurred in the late Mesozoic Era, so it seems interesting to compare theparameters of ultradian and infradian biorhythms in phylogenetically distant insectivorous mammals and rodents to identify common mechanisms for their formation.

The aim of the study is to identify ultradian and infradian rhythms in the dynamics of testosterone concentration in the blood serum of white-breasted hedgehogs.

## Results

### Ultradian rhythms of testosterone concentration in the blood of the white-breast hedgehogs

**Figure 1:**
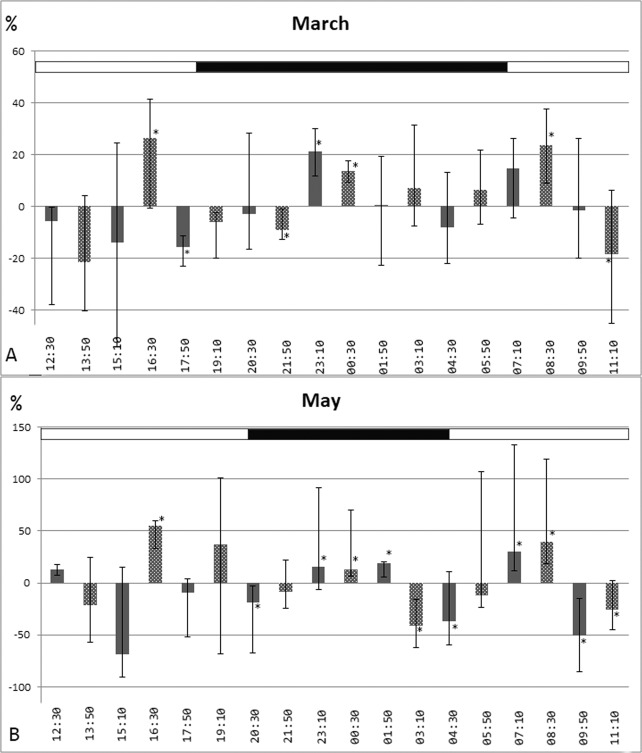
The daily dynamics of testosterone concentration in serum of hedgehogs. Data were normalised by calculating the deviations from the individual’s mean level and plotted as group mean in the sampling-period of (**A**) March 7–8, 2019 (n = 7, with in-phase rhythm) and (**B**) May 10–11, 2018 (n = 6, with in-phase rhythm). The medians and interquartile range are represented. In both March and May, the maximum values of the studied indicator were detected in the period of 7:10–8:30, 15:10–16:30 and 23:10–00:30.

On March 7–8, 2019, when the sunset was at about 18:20 and sunrise was at 7:00, we studied individual fluctuations of testosterone concentration in the blood of 9 hedgehogs by the method of cosinor-analysis. We revealed 8-hour in-phase fluctuations in 7 out of 9 animals with maximums at 7:10–8:30, 15:10–16:30 and 23:10–00:30 (Fig. 1A, Suppl 1). However, in one hedgehog, the acrophase of the 8-hour rhythm was observed 120 min earlier (Table 1, Suppl 1). In another hedgehog, the maxima of testosterone concentration were observed at 16:30 and at 8:30 of the next day; thus, a 16-hour interval was between 2 maxima.

**Table 1:** Parameters of ultradian individual rhythms of testosterone concentration in hedgehogs on March 7–8, 2019 (Cosinor-analysis).

Number of animal	Period, hours	Mean level of testosterone concentration in blood per 24 h, nmol/l	Amplitude of testosterone concentration in blood, nmol/l	Acrophase, time of a day from the beginning of the experiment (March 7, 2019, 8:30)
№3	16	65.5	14.5	16:30, 8:30
№4	8	56.3	19.5	15:10, 23:10, 7:10
№6	8	65.6	14.4	12:30, 20:30, 4:30
№7	8	59.0	15.0	15:10, 23:10, 7:10
№8	8	38.6	16.1	8:30, 16:30, 0:30
№9	8	26.4	13.3	15:10, 23:10, 7:10
№10	8	63.5	16.6	8:30, 16:30, 0:30
№11	8	78.5	16.1	8:30, 16:30, 0:30
№12	8	52.1	12.9	8:30, 16:30, 0:30

In May 10–11, 2018, when the sunset was at about 20:25 and sunrise was at 4:30, 4 out of 7 animals had an 8-hour in-phase rhythm of testosterone concentration with acrophases at 7:10–8:30, 15:10–16:30 and 23:10–00:30 (Fig. 1B, Suppl 1) at the same times as in March. There were 2 animals which showed 2 maxima at 16:30 and 8:30 (again similar to March), i.e. there were 16 hours between the daily maximum and the one the next morning (Table 2); 1 hedgehog only showed 1 maximum at 20:30 (Suppl 1).

**Table 2:** Parameters of ultradian individual rhythms of testosterone concentration in hedgehogs in May 10–11, 2018 (Cosinor-analysis).

Number of animal	Period, hours	Mean level of testosterone concentration in blood per 24 h, nmol/l	Amplitude of testosterone concentration in blood, nmol/l	Acrophase, time of a day from the beginning of the experiment (May 10, 2018, 12:30)
№1	24	9.4	2.2	20:30
№2	8	17.3	5.1	16:30, 0:30, 8:30
№4	16	23.9	8.3	16:30, 8:30
№5	8	10.2	2.9	16:30, 0:30, 8:30
№6	8	18.5	4.9	15:10, 23:10, 7:10
№8	8	12.9	3.8	15:10, 23:10, 7:10
№9	16	11.3	2.8	16:30, 8:30

Thus, an 8-hour rhythm of testosterone concentration was detected in 12 of 16 cases, and in-phase 8-hour rhythm was detected in 11 cases. Moreover, in the 3 hedgehogs, 16 hours were between the testosterone maxima, and these maxima coincided with the acrophase time points of an 8-hour biorhythm. It should be noted that on the basis of a 24 h study duration, we can’t approve the existence of a 16-hour rhythm of testosterone concentration dynamic. Only the 1 animal showed a low-amplitude daily rhythm of the studied parameter.

In Fig. 1, the daily dynamic percentage deviation of testosterone concentration from the individual average level in the period of March 7–8, 2019 (n = 7) and May 10–11, 2018 (n = 6) is presented. We excluded 3 animals from the analysis of data: 2 hedgehogs studied in the period of March 7–8 and 1 animal in the period of May 7–8 due to the lack of an 8-hour rhythm or the difference in its phase from most animals.

In each experiment, 2 subgroups of animals were formed. In order to avoid possible synchronisation of ultradian rhythms, with a stressful effect from the first blood collection, the sampling of the second group started with a 240 minute delay after the first group. The dynamics of the testosterone level in the first and second subgroups were similar, suggesting the absent of connection between the phase of the ultradian rhythm and the moment of the first stress effect caused by blood sampling.

The total testosterone concentration of all hedgehogs in the acrophase and bathyphase of the 8-hour rhythm significantly differed among themselves in both March (Table 3) and May (Table 4).

**Table 3:** The concentrations of testosterone in the acrophase and the bathyphase of the 8-hour rhythm in all animals in the group studied on March 7–8, 2019 (n = 9).

Phase	Time of a day, h	Testosterone level, nmol/l (Me; Q25-Q75)	Statistical significance, p (Mann-U test)
Acrophase	23:10–00:30 7:10–8:30 15:10–16:30	70.2(55.3;76.5) n = 27	p = 0.0016
Bathyphase	01:50–5:50 9:50–13:50 17:50–21:50	52.3 (36.1; 63.3) n = 27

**Table 4:** The concentrations of testosterone in the acrophase and the bathyphase of the 8-hour rhythm in all animals in the group studied on May 10–11, 2018 (n = 7).

Phase	Time of a day, h	Testosterone level, nmol/l (Me; Q25-Q75)	Statistical significance, p (Mann-U test)
Acrophase	23:10–00:30 7:10–8:30 15:10–16:30	18.1(10.3;22.0) n = 21	p = 0.023
Bathyphase	01:50–5:50 9:50–13:50 17:50–21:50	10.0 (5.8; 16.8) n = 21	

It is noticeable that, in both cases, the maximum values of the studied indicator were detected in the period 7:10–8:30, 15:10–16:30 and 23:10–00:30. Thus, the maximum values of testosterone concentration are recorded at the same hours of the day despite the day length (11.25 hours at the beginning of March and 16.0 hours at the beginning of May). It should be noted that at 19:10, only in May, an increase in testosterone levels was detected in 2 animals that may be associated with an evening decrease in illumination (sunset is at 20:25). However, the testosterone concentration at this time point has a large scatter and does statistically not differ significantly from any research points.

### Infradian rhythms of testosterone concentration in the blood of the white-breast hedgehogs

**Figure 2:**
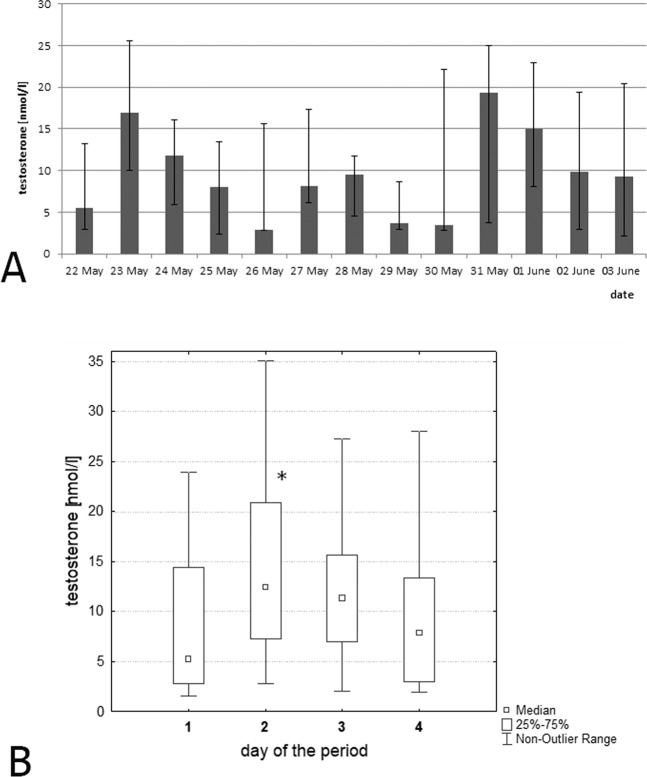
(**A**) Dynamics of serum testosterone concentration in the group of hedgehogs (n = 7) obtained in the period from May 22 to June 3, 2018. Blood samples were taken from 7:40 to 8:50. (**B**) Distribution of all obtained data of testosterone concentration by days of the 4-day period. The first day of the 4-day period included getting data on May 22, 26, 30 and June 3; the second, on May 23, 27 and 31; the third, on May 24 and 28 and June 1 and the fourth, on May 25 and 29 and June 2. The concentrations of testosterone in acrophase (the 2nd day) and bathyphase (the 4th day) were significantly different between each other, p = 0.027.

Between May 22 to June 3, 2018, testosterone concentration was daily measured at 7:40–8:50, and we detected a 4-day rhythm of testosterone level (Fig. 2A). The maxima were observed on May 23, May 27–28 and May 31, and the minimums were found on May 22, May 26, May 29–30 and June 2–3. In order to identify the statistical significance of this rhythm, all the obtained indicators were distributed by day over the 4-day period (Fig. 2B). The first day of the 4-day period included data getting on May 22, 26, 30 and June 3; the second, on May 23, 27 and 31; the third, on May 24, 28 and June 1 and the fourth, on May 25, 29 and June 2. It turned out that testosterone concentration on the second day of the 4-day period (in May 23, 27 and 31) was statistically significantly higher than its values on the first and the fourth day with p = 0.005 and p = 0.027, respectively. Thus, we established a 4-day rhythm in the dynamics of the serum testosterone concentration obtained at 7:40–8:50 in the morning.

**Figure 3:**
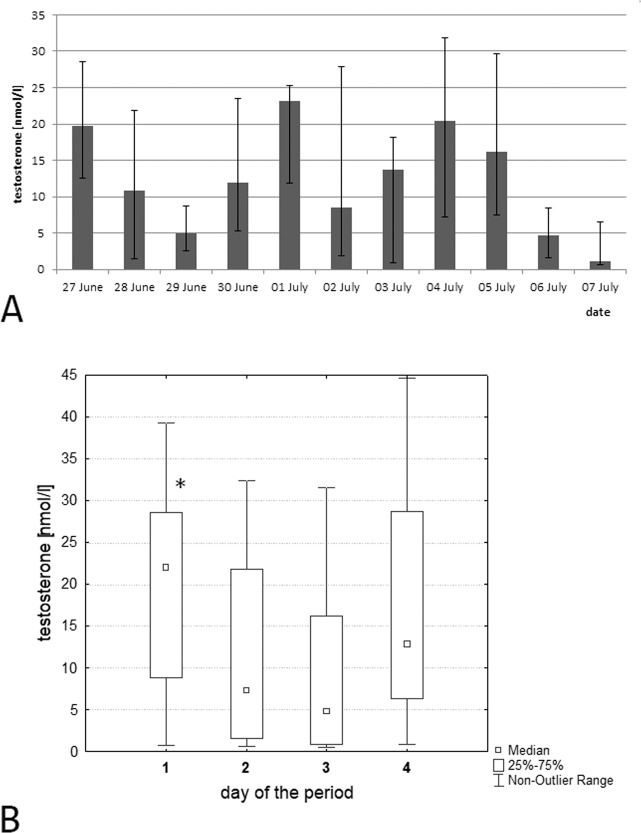
(**A**) The dynamics of serum testosterone concentration in the group of hedgehogs (n = 6) in the period from June 27 to July 7, 2018. Blood samples were taken from 16:15 to 16:50. (**B**) Distribution of all obtained indicators of testosterone concentration by days of the 4-day period. The first day of the 4-day period included data getting in June 27, July 1 and 5; the second, on June 28 and July 2 and 6; the third, on June 29 and July 3 and 7 and the fourth, on June 30 and July 4. Differences of testosterone concentration are significant on the first and the third day of the 4-day period, p = 0.013.

After that, we conducted the daily testosterone concentration study in the evening (16:30). A 4-day rhythmicity was also observed during this period with maxima on June 27, July 1, 4–5 and minimum on June 29, July 2–3 and July 6–7 (Fig. 3A). All obtained data were also distributed by day of the 4-day period (Fig. 3B). The values of testosterone in the acrophase of the 4-day rhythm (June 27, July 1 and 5) were 22.0 (8.9; 28.6) nmol/l, and in the bathyphase (June 29, July 3 and 7) were 4.8 (0.9; 16.2) nmol/l. The concentrations of testosterone in acrophase (the first day) and bathyphase (the third day) were statistically significantly different between each other, p = 0.013 (Fig. 3B). Thus, the 4-day testosterone concentration rhythm is also detected when taking blood in the evening (16:15–16:50).

Thus, according to an individual analysis of infradian rhythms of testosterone concentration by the method of cosinor-analysis from May 22 to June 3, 2018, all animals had a 4-day rhythm. In 5 animals, the acrophase of rhythm occurred on May 23, 27 and 31 (Suppl 1). In one animal, the maximum values were observed a day earlier and, in the another, a day later than in most hedgehogs (Table 5).

**Table 5:** Parameters of infradian individual rhythms of testosterone concentration in hedgehogs on May 22 to June 3, 2018 (Cosinor-analysis).

Number of animal	Period, days	Mean level of testosterone concentration in blood per 4 d, nmol/l	Amplitude of testosterone concentration in blood, nmol/l	Acrophase, data from the beginning of the experiment (May 22, 2018)
№1	4	7.4	2.1	May 23, 27, 31
№2	4	19.1	4.7	May 24, 28; June 1
№4	4	8.3	7.0	May 23, 27, 31
№6	4	6.4	2.4	May 22, 26, 30; June 3
№7	4	18.9	9.8	May 23, 27, 31
№8	4	11.8	6.8	May 23, 27, 31
№9	4	8.2	5.1	May 23, 27, 31

Between June 27 and July 7, 5 out of 6 animals showed a 4-day period in the dynamics of serum testosterone concentration, and 1 hedgehog had a 3-day period (Table 6). In one animal, the maximum level was observed a day earlier than in most of the studied hedgehogs (Suppl 1).

**Table 6:** Parameters of infradian individual rhythms of testosterone concentration in hedgehogs on June 27 to July 7, 2018 (Cosinor-analysis).

Number of animal	Period, days	Mean level of testosterone concentration in blood per 4 d, nmol/l	Amplitude of testosterone concentration in blood, nmol/l	Acrophase, data from the beginning of the experiment (June 27, 2018)
№2	4	21.0	8.7	June 27; July 1, 5
№3	4	6.5	8.3	June 27; July 1, 5
№4	4	5.3	3.2	June 27; July 1, 5
№7	3	31.9	4.9	June 27; July 1, 5
№8	4	16.1	9.3	June 27; July 1, 5
№9	4	10.9	7.8	June 30, July 4

Thus, the parameters of the individual rhythms of animals in most cases coincide with those of the group as a whole. However, some individuals have phase deviations for 1 day. The 3-day rhythm revealed in 1 of 13 cases. In our opinion, this case cannot be considered regular because this animal showed a 4-day rhythm in the first experiment in May, 2018.

**Figure 4:**
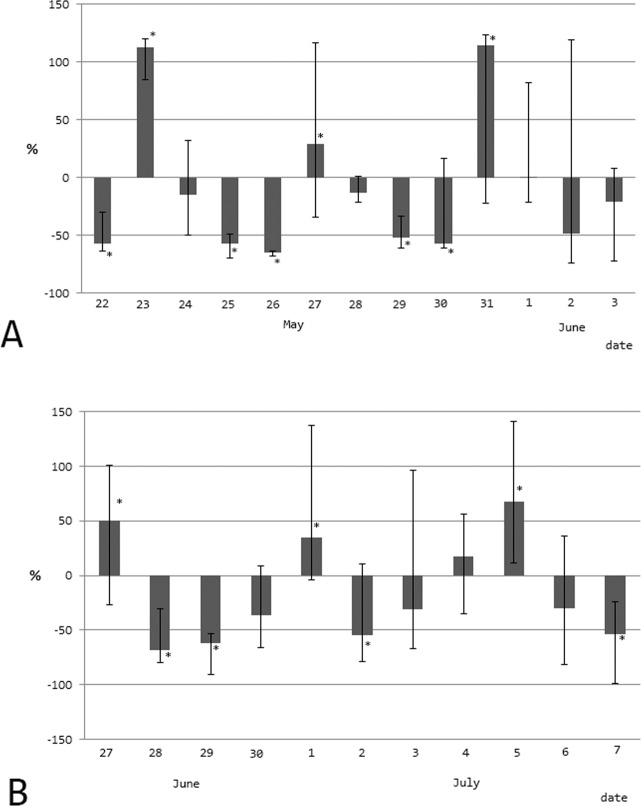
Multiday dynamics of the percentage deviation of testosterone concentration in serum from the individual average level in hedgehogs in the period (**A**) May 22–June 3, 2018 (n = 5 with in-phase rhythm) and (B) June 27–July 7, 2018 (n = 4, with in-phase rhythm). The medians and interquartile range are represented.

Figure 4 shows the dynamics of the percentage deviation of the concentration of testosterone from the individual average level in hedgehogs in the period of May 22 to June 3, 2018 (n = 5, showed in-phase rhythm) and in the period in June 27 to July 7, 2018 (n = 4, showed in-phase rhythm). So the animals with only a 4-day in-phase biorhythm were included in this analysis. The 1 animal from the first experiment and 2 animals from the second experiment were excluded. Using of the calculating autocorrelation method between the original series and the series shifted on 1, 2, 3 and 4 days, we revealed the reliability of the 4-day rhythm in both cases (Tables 7 and 8).

**Table 7:** The calculated autocorrelation coefficients for the dynamics of the testosterone concentration in hedgehogs on May 22 to June 3, 2018 (n = 5).

Shifting, days	r	p
1	0.066	0.864
2	−0.309	0.455
3	−0.250	0.588
4	0.942	**0.004**

**Table 8:** The calculated autocorrelation coefficients for the dynamics of the testosterone concentration in hedgehogs on June 27 to July 7, 2018 (n = 4).

Shifting, days	r	p
1	−0.090	0.802
2	−0.612	0.059
3	−0.018	0.960
4	0.916	**0.001**

## Discussion

We found an 8-hour ultradian rhythm of testosterone levels in most hedgehogs’ blood serum. The maximum testosterone concentrations in the serum of hedgehogs are recorded in the periods of 7:10–8:30, 15:10–16:30 and 23:10–00:30, independent of photoperiod (11.25 or 16.0 hours). Consequently, the peaks of testosterone concentration in hedgehogs are not dependent on the time of the sunrise and sunset or light mode. In experimental works, it was also found that even the conditions of constant lighting, leading to a change in the period of the circadian rhythm, do not change the duration of ultradian biorhythms^27,28^.

Thus, the phase of some ultradian biorhythms, including the dynamics of the concentration of testosterone in the blood serum, is not determined by the light/dark mode. Perhaps this 8-hour rhythm of testosterone is needed to synchronise sperm maturation processes^3^, whose frequency is not affected by the duration of the circadian rhythm^29^.

In our previous work, we showed an about-8-hour periodicity in the dynamics of testosterone concentration in rats^18^. We have shown that, in rabbits, the inversion of the light mode or the shift of the light period relative to the local time did not lead to a change in the acrophase of the 8-hour testosterone rhythm. Moreover, a month after the trans-meridian movement of rabbits from Omsk to Moscow while maintaining the light mode characteristic of Omsk, testosterone concentration peaks were detected at midnight, 8:00 and 16:00 local Moscow time (whereas the time differences between the sunset/sunrise in Moscow and Omsk is 3.5 h)^19^. This fact points to a possible existence of an external synchroniser for ultradian rhythms.

Ultradian rhythms with the same period and phase are also found in humans. The maximum number of calls to ambulance teams for myocardial infarction is noted at midnight, 8:00 and 16:00^30^. The author connects the development of acute coronary syndrome, which often leads to myocardial infarction, with the activation of the sympathoadrenal system during these hours.

In one work^25^, studying seasonal testosterone rhythms in hedgehogs, blood samples were taken at the same fixed time relative to sunset/sunrise—5 hours after the sunrise and 2 hours after the sunset every month for a year. The testosterone concentration in the morning and evening blood sampling in the vast majority of cases were comparable. However, the concentration of testosterone in April in the evening hours was 2 times higher than in the morning hours, and in July, on the contrary, 2 times lower. This can probably be explained by blood sampling in different phases of ultradian rhythms. We have shown the ultradian rhythm of testosterone concentration is not associated with the time of sunset/sunrise but is timed to a definite time of day. Thus, blood sampling relative to the setting of the Sun will lead to a gradual shift in blood sampling relative to the ultradian rhythm phase of this hormone. So, if studies of individual or seasonal testosterone levels in animals are conducted, the phase of ultradian biorhythms will need to be taken into account.

When we studied the ultradian rhythms of testosterone fluctuations in March, a 16-hour change in testosterone concentration was detected in only 1 animal. Whereas in an experiment conducted in May, a 16-hour period between the maxima of testosterone concentration was found in 2 animals, and in another one, a 24-hour period was detected. We believe that this is due to the average concentration of testosterone, which in March, was 59.0 (52.1; 65.5) nmol/l and in May, was 11.3 (9.4; 18.5) nmol/l. It is likely that at the beginning of the breeding season, when the concentration of testosterone is at its maximum, higher-frequency rhythms prevail. While at the end of the breeding season the level does not increase in every acrophase of the 8-hour rhythm in some animals. Just like in rabbits, immediately after puberty, a 4-hour rhythm of testosterone is observed, and in adult animals, an 8-hour rhythm^19^.

Our study of the infradian rhythm of testosterone levels in hedgehogs showed the presence of about a 4-day rhythm. In humans, a 4-day rhythm was found in boys^31^ and men aged 27–33 years^32^ in the dynamics of testosterone concentration. It is important to note that 1 man had a stable 8-day rhythm of this hormone. A 4-day rhythm of fluctuations in the concentration of testosterone and corticosterone was revealed in male Wistar rats^18^.

Probably, a 4-day infradian biorhythm is a characteristic of most mammals. The acrophase of the fluctuations in the corticosterone and testosterone concentrations in rats and humans shifts to a day ahead, every 60–73 days. So the exact period of infradian biorhythm is 4.058 days. The Earth year includes 90 4.058-day periods, that allows the predicting of the phase of this period. The acrophase of the 4-day rhythm of corticosterone and testosterone concentrations in nocturnal species appears a day earlier. In daytime species, it coincides with the bathyphases of the 12-day rhythm of oesophagus epithelium mitotic activity, which was presented in^33^. It is necessary to clarify that the maximum serum testosterone values in rats are observed in the evening because of their nocturnal activity, but in humans with daily activity, they are observed in the morning hours. In this regard, the acrophase of the 4-day testosterone rhythm in rats occurs in the evening, 12 hours before the maximum indicator of this hormone is observed in humans in the morning. The dates of maximum testosterone concentration of hedgehogs detected in this work in the morning hours on May 23, 27 and 31 correspond to peaks calculated for humans, and the testosterone values determined in the evening hours on June 27, July 1 and July 4–5 correspond to data predicted for rats. Thus, the period of the infradian rhythm of testosterone is the same in hedgehogs, rats and humans, and its phases probably coincide.

It is known that, in rodents, the smell of urine secreted by a female during oestrus and even dioestrus affects the sexual behaviour of males nearby and influences the level of hormones in the blood, including testosterone^34,35^. We do not exclude the possibility of the presence of wild individuals of related species, for example, the European hedgehog, in the study area. However, in the experiment conducted on March 7–8, any influence of the smell of females is excluded; during this period, wild hedgehogs were still sleeping (in 2019 they woke up in the first 10 days of April). In late June–early July, when we conducted the study, hedgehogs had already had offspring. We did not find any data about the exact duration of the hedgehogs’ oestrous cycle of the genus *Erinaceus*, but it is known that the length of the oestrous period in the long-eared hedgehogs (*Hemiechinus auritus*) is 7.9 days^36^.

Consequently, the infradian rhythm of testosterone in male hedgehogs is exactly 2 times shorter than the duration of the oestrous cycle of their females. That indicates their relationship and mutual synchronisation. It could be assumed that, in rats, the 4-day oestrous cycle of females through the olfactory channel determines a similar rhythm of testosterone. However, a 4-day biorhythm is also observed in males that are isolated from the smell of their sexual partners^32^, which indicates the absence of a female’s influence in the generation of a 4-day rhythm of testosterone in male rats. Moreover, the 4-day rhythm of testosterone was also found in men^31^, while in women, the menstrual cycle is much longer.

We did not find any works of other research groups where authors would purposefully search for external synchronisers of ultradian and infradian rhythms. However, a possible factor synchronising ultradian and infradian biorhythms are quasi-rhythmic changes in the geomagnetic field^37,38^. Our studies can be as a key to the search for an external biotrophic factor that determines the results obtained in this work.

Thus, the parameters of ultradian and infradian biorhythms of testosterone concentration in phylogenetically distant insectivorous mammals and rodents are similar; therefore, the mechanisms of formation of these biorhythms are most likely the same and were formed before the separation of these superorders had occurred in the late Mesozoic Era.

## Conclusion

1. There is an ultradian 8-hour rhythm in serum testosterone in most hedgehogs.
2. Peak time of testosterone concentration is independent from sunrise/sunset time and it has maxima in 7:10–8:30, 15:10–16:30 and 23:10–00:30.
3. There is a 4-day infradian rhythm of testosterone concentration in most hedgehogs.

## Methods

Studies were carried out on 12 male white-breasted hedgehogs *Erinaceus roumanicus* (Table 9). Hedgehogs were captured by hand in the Spassky district of the Ryazan region. The hedgehogs were kept in an open-air cage with a total area of 80 m^2^ in the summer in the territory of the Chernogolovka biological station, the Joint Usage Centre’s ‘Collection of live mammals’, A.N.Severtsov Institute of Ecology and Evolution RAS. From autumn to spring, the animals were in an unheated room in pens of 1 m^2^ with natural light. Daily hedgehogs received minced meat from raw chicken with bones with the addition of raw eggs. All manipulations with animals were carried out according to the European convention for the protection of vertebrate animals used for experimental and other scientific purposes, Strasbourg, 1986. The study received permission from the Bioethics Committee of A.N. Severtsov Institute of Ecology and Evolution RAS No 14 dated1/15/2018.

**Table 9:** Numbers of hedgehogs using at the studying of infradian and ultradian rhythms.

Number of animal	Studying of Infradian Rhythms		Studying of Ultradian Rhythms	
	May–June 2018	June–July 2018	May 2018	March 2019
1	+	* Low testosterone level 1.24 ± 0.6 nmol/l	+	Died
2	+	+	+	Died
3	* Low testosterone level 1.12 ± 0.5 nmol/l	+	* Low testosterone level 0.95 ± 0.4	+
4	+	+	+	+
5	Abscess	-	+	Died
6	+	* Low testosterone level 0.90 ± 0.3 nmol/l	+	+
7	+	+	Low body weight (790 g)	+
8	+	+	+	+
9	+	+	+	+
10	it was caught later	it was caught later	it was caught later	+
11	it was caught later	it was caught later	it was caught later	+
12	it was caught later	it was caught later	it was caught later	+
Number of animals using at the experiments (blood sampling)	8	8	8	9
Number of animals. whose testosterone level data were used in statistical analysis and graphing	7	6	7	9

Notes. * This low testosterone concentration is not typical for males in the reproductive period and corresponds to females. That does not allow the identification of any rhythms. Median (Q25–Q75) of testosterone concentration of all animals using at the experiments in May–June is 11.3 (3.1; 17.3) nmol/l, in June–July is 14.2 (1.8; 25.3) nmol/l. We excluded all animals which testosterone values deviated by more than 90% from the group mean.

The study of ultradian rhythms of testosterone dynamics was performed in 2 time intervals; the first interval of studying was from 8:30 in March 7 to 9:50 in Match 8, 2019 with a day length of 11 hours 15 minutes. And the second period was from 12:30 in May 10 to 13:50 in May 11, 2018 with a day length of 16 hours (Table 9). There were 2 groups of animals formed. Blood of animals from each group was taken every 160 minutes. However, the 2 groups were sampled with a time shift resulting in a daily curve with an interval of 80 minutes. In order to avoid possible synchronisation of ultradian rhythms with a stressful effect from the first blood collection, the sampling of the second group started with a 240 minutes delay after the first group.

We took blood samples daily at 7:40–8:50 from May 22 to June 3, 2018 and at 16:15–16:50 from June 27 to July 7, 2018 in order to study of infradian rhythms (Table 9).

Blood sampling was performed from the femoral veins using a 2 ml syringe with a 0.7 × 35 mm needle, following a modification of the indicators by Lewis *et al*.^39^. The volume of each single blood sample was 150–300 μl; therefore, for the entire period of the study, the hedgehog lost no more than 2–3 ml of blood. During the experiment (when we had been taking blood samples), no hedgehog hadn’t died. There were 3 hedgehogs that died in August, probably due to high air temperature. The blood was centrifuged 30 minutes after collection and the obtained serum was stored for no more than a month at a temperature of −20 °C.

We used Zoletil at a dose of 10 mg/kg body weight for anaesthesia (Virbac Sante Animale, France). The 10 mg/kg of Zoletil is not a high dose. According to the prescription, this dose is used for clinical examination and quick surgical interventions in veterinary science. Using this dose, we were able to deploy hedgehogs for blood sampling. This condition of animals lasted only 10–15 minutes, and after that, the hedgehogs could curl up.

The serum testosterone concentration was determined by ELISA using “Immunotech” kits (Russia, Mosсow) according to an attached instruction. The sensitivity of the method is 0.18 nmol/L. Testosterone concentrations were studied in duplicates and we used their average value for further analysis. If a coefficient of variation had been more than 5%, we repeated the measurements. The cross- reactivity of antibodies to testosterone is 9% for 5-dihydrotestosterone, 1% for 11-hydroxytestosterone, 1% for 5-androsten-3.17-diol and less than 0.1% for all other tested steroids. The colour reaction was recorded on a multichannel ELISA reader ANTHOS 2010, Austria.

Statistical data processing was performed using the Statistica Ultimate Academic 13 for Windows software package. The data obtained were expressed as the median and interquartile ranges of Me (Q25- Q75). To identify periods of infradian rhythms, the autocorrelation coefficient between the original series and the series shifted by 1, 2, 3 and 4 days was calculated. The statistical significance of differences in acrophase (the period point when the indicator has maximum values) and bathyphase (the period point when the indicator has minimum values) was evaluated using the non-parametric Mann-Whitney test and the Kruskal-Wallis multiple comparison test. To identify individual rhythms of the testosterone concentration dynamics, we used the method of cosinor-analysis in the Cosinor-Analysis 2.4 for Excel 2000/XP. Cosinor-analysis is a short time series processing method. It is based on the approximation of a time series by a cosine wave. The approximation is done by the least squares method.

## Supplementary information

Supplementary information.
